# Activity of DNA- and RNA-Guided Prokaryotic Argonautes in Human Mitochondria

**DOI:** 10.3390/cells15121129

**Published:** 2026-06-22

**Authors:** Beatrisa Rimskaya, Ekaterina Kropocheva, Iaroslava Ponomareva, Lada Karchemkina, Lidiya Lisitskaya, Daria Gelfenbein, Egor Ulashchik, Vadim Shmanai, Andrey Kulbachinskiy, Ilya Mazunin

**Affiliations:** 1 Moscow Center for Advanced Studies, Moscow 123592, Russia; yarosha1810@gmail.com (I.P.); lada.karchemkina@yandex.ru (L.K.); 2 The Center for Bio- and Medical Technologies, Moscow 121205, Russia; katerinakropocheva@yandex.ru; 3 Institute of Gene Biology, Russian Academy of Sciences, Moscow 119334, Russiaes_dar@inbox.ru (D.G.); avkulb@yandex.ru (A.K.); 4 Institute of Physical Organic Chemistry, National Academy of Science of Belarus, 220072 Minsk, Belarus; e.ulashchik@gmail.com (E.U.); v.shmanai@gmail.com (V.S.); 5 Department of Biology and Genetics, Petrovsky Medical University, Moscow 119435, Russia

**Keywords:** prokaryotic argonautes, mitochondria, D-loop, mtDNA copy number

## Abstract

**Highlights:**

**What are the main findings?**

- Prokaryotic Argonaute proteins are efficiently imported into human mitochondria using the Su9 targeting sequence.
- DNA-guided Argonautes are inactive in cells, highlighting guide delivery as a key limitation.

**What are the implications of the main findings?**

- RNA-guided DecAgo exhibits catalytic activity in mitochondria and reduces mtDNA copy number.
- mtDNA depletion by DecAgo occurs independently of exogenous guide delivery.

**Abstract:**

Precise manipulation of mitochondrial DNA (mtDNA) by CRISPR-Cas systems remains challenging, largely due to inefficient import of guide RNAs, motivating the exploration of alternative programmable nucleases. Here, we show that prokaryotic Argonaute nucleases (pAgos) of various classes can be efficiently targeted to human mitochondria. Using the Su9 mitochondrial targeting sequence from *Neurospora crassa*, we achieved robust mitochondrial import of four pAgos—DecAgo, CbuAgo, KmaAgo and RslAgo. As a functional readout of their activity in cells, we targeted the single-stranded D-loop region, which plays a central role in mtDNA replication and maintenance, reasoning that cleavage at this site was expected to potentially result in a reduction in mtDNA copy number. Of the four enzymes, only RNA-guided DecAgo induced a pronounced reduction in mtDNA levels, decreasing copy number approximately fivefold within 48 h. Unexpectedly, this effect occurred independently of exogenous guides, suggesting that DecAgo may utilize endogenous mitochondrial guide RNAs. These findings identify DecAgo as an active nuclease in human mitochondria and reveal a previously unrecognized mode of targeting, highlighting the need to further investigate the underlying mechanism and the potential role of endogenous guide molecules, as well as improving targeting specificity.

## 1. Introduction

Mitochondria are double-membrane organelles responsible for cellular energy production, metabolic regulation, and apoptosis signaling. Mitochondria possess their own genome (mtDNA), which encodes key components of the oxidative phosphorylation machinery and is inherited independently of nuclear DNA. Human mtDNA is a compact, circular molecule of approximately 16.5 kb, present in multiple copies per cell, and consists of a heavy (H) and light (L) strand. It contains a non-coding regulatory region known as the displacement loop (D-loop), which harbors essential elements controlling mtDNA replication and transcription, including the origin of replication for the H-strand and transcription promoters [1]. The D-loop forms a triple-stranded DNA structure and is partially single-stranded due to the presence of a short nascent DNA strand. In addition, transcription of the control region generates RNA molecules that can hybridize to the complementary DNA strand, forming RNA-DNA hybrids (R-loops) that displace the opposing DNA strand. These structures represent a distinct triple-stranded configuration and are proposed to play a central role in the initiation and regulation of mtDNA replication [2].

Early attempts to manipulate mtDNA relied on protein-based programmable nucleases, including zinc-finger nucleases (mtZFNs) and transcription activator-like effector nucleases (mitoTALENs), which enabled selective elimination of mutant mtDNA molecules through targeted double-strand breaks [3,4]. These approaches demonstrated that the levels of heteroplasmy (the presence of both mutant and wild-type mtDNA within the same cell) can be shifted by selective degradation of mitochondrial genomes. However, nuclease-based strategies remain ineffective in homoplasmic contexts, where all mtDNA copies carry the pathogenic variant such as Leber’s hereditary optic neuropathy (LHON) [5]. These limitations highlight the need for approaches that enable direct correction of pathogenic variants. CRISPR-Cas systems first revolutionized nuclear genome engineering as programmable RNA-guided nucleases. However, direct application of classical CRISPR-Cas nucleases to mtDNA has remained challenging, primarily because efficient import of guide RNAs into mitochondria has not been reliably achieved [6,7]. This limitation stimulated the development of RNA-free mitochondrial editing strategies. In particular, base-editing systems derived from bacterial cytidine deaminases, such as DddA-derived cytosine base editors (DdCBEs), provided the first programmable tools for direct nucleotide conversion in mtDNA without double-strand breaks [8,9].

Although several studies have reported partial import of RNA molecules into mitochondria, efficient delivery remains constrained by RNA length and structural requirements [10,11,12]. These limitations motivate the exploration of alternative programmable nucleases that rely on short nucleic acid guides. Prokaryotic Argonaute proteins (pAgos) represent a promising class of programmable nucleases capable of using short nucleic acid guides to direct sequence-specific cleavage of DNA or RNA targets. Depending on their structure, different pAgos can utilize DNA or RNA guides, and recent studies have identified mesophilic variants with catalytic activity at physiological temperatures [13]. Unlike CRISPR-Cas nucleases, pAgos do not require a protospacer adjacent motif (PAM) for target recognition, providing greater flexibility in sequence targeting. In addition, pAgos are typically more compact than Cas nucleases (~70–100 kDa compared to ~160 kDa for Cas9), which may facilitate their delivery into mitochondria.

Over the past decade, multiple studies have demonstrated DNA cleavage by bacterial and archaeal Argonautes in vitro, as well as guide-dependent DNA interference mechanisms in prokaryotes, suggesting their potential role in host defense against mobile genetic elements, phages and invading nucleic acids [14]. In addition to pAgos targeting DNA, a distinct class of Argonautes has been identified that mediate DNA-guided RNA cleavage [15,16,17]. Recent studies demonstrate that eukaryotic Argonautes can be harnessed for RNA manipulation, including programmable RNA editing and repair of point mutations [18]. Engineered applications of Argonautes have also been explored for genome editing and nucleic acid detection; however, their functionality in mammalian cells remained largely uncharacterized [19]. Given that pAgos preferentially target multicopy genetic elements in vivo and introduce single-stranded cleavage events in nucleic acid substrates, mitochondrial DNA may provide a particularly suitable substrate for their activity. In addition to its multicopy nature, mtDNA contains single-stranded DNA intermediates within the D-loop region associated with replication, features that are largely absent from the double-stranded nuclear genome. In our previous work, we demonstrated that the RNA-guided DNA-targeting pAgo protein from *Alteromonas macleodii* (AmAgo) can be targeted to mammalian mitochondria and decrease the mtDNA copy number, suggesting that these nucleases may provide a foundation for mitochondrial genome manipulation [20].

To extend these findings and broaden our understanding of pAgo functionality in mitochondria, here we investigated additional pAgos, including DecAgo from *Deinococcus cavernae* (RNA-guided DNA-targeting pAgo) [21], CbuAgo from *Clostridium butyricum* [22], KmaAgo from *Kurthia massiliensis* (DNA-guided DNA-targeting pAgos) [23,24], and RslAgo from *Runella slithyformis* (DNA-guided RNA-targeting pAgo) [16,22]. All four proteins exhibit programmable nuclease activity in vitro and are active under physiological conditions. These enzymes differ in guide and target preferences and catalytic efficiency, making them suitable candidates for comparative evaluation in a mitochondrial environment. However, whether such nucleases can functionally operate within human mitochondria and influence mtDNA maintenance has remained unknown.

Here, we showed efficient mitochondrial import of all four tested pAgos; however, among them, only DecAgo significantly reduced the mtDNA copy number. Notably, this effect occurred independently of exogenous guide delivery, suggesting that DecAgo may utilize endogenous mitochondrial nucleic acids as guides. These findings identify DecAgo as an active nuclease in human mitochondria and highlight Argonaute proteins as a promising alternative platform for mitochondrial genome manipulation.

## 2. Materials and Methods

### 2.1. Molecular Cloning

Lentiviral transfer plasmids were generated using a modified pUltra backbone (Addgene plasmid #24129, Watertown, MA, USA) following standard molecular cloning procedures. Assembly of mitochondria-targeted prokaryotic Argonaute (pAgo) constructs was performed in two sequential cloning steps.

In the first step, the GFP expression cassette of the pUltra vector was replaced with DNA fragments encoding either a 3×FLAG-mitochondrial targeting sequence (MTS) derived from subunit 9 of *Neurospora crassa* ATP synthase (Su9), selected based on previously reported mitochondrial import efficiency [20], or a 3×FLAG tag alone, generating intermediate recipient plasmids. In the second step, coding sequences of individual pAgos were PCR-amplified and inserted downstream of the 3×FLAG-MTS or 3×FLAG cassette. For DecAgo, RslAgo, and KmaAgo constructs, cloning was performed using NheI and AsiGI restriction sites, whereas the CbuAgo construct was assembled using BamHI and NheI sites. All inserts were introduced by restriction-ligation cloning. All constructs were verified by Sanger sequencing. Primer sequences used for amplification are listed in Supplementary Table S1.

### 2.2. Protein Expression and In Vitro Analysis

Wild-type and catalytically inactive pAgo proteins were expressed in *E. coli* BL21(DE3) cells harboring pET28-based expression constructs as described previously [16]. Briefly, cultures were grown in LB medium supplemented with kanamycin (50 μg/mL) at 37 °C to mid-log phase, cooled to 16 °C, induced with 0.1 mM IPTG, and incubated overnight at 16 °C prior to harvest. Cell pellets were lysed by sonication, and recombinant proteins were purified using Ni^2+^-affinity chromatography followed by cation-exchange chromatography. Purified fractions were concentrated by ultrafiltration, supplemented with glycerol (50%), and stored at −20 °C or −80 °C.

Guide RNAs/DNAs and target DNA oligonucleotides were synthesized as previously described [16,20]. In addition to unmodified and phosphorylated guides, chemically modified guide RNA/DNA variants were synthesized, including OMe/PS-modified guides, 5′-vinylphosphonate-containing guides, and guide RNAs labeled with Alexa Fluor 488 via post-synthetic conjugation. Nuclease activity was assessed in vitro using synthetic substrates in reaction buffer (10 mM Tris-HCl pH 7.9, 100 mM NaCl, 5 mM MgCl_2_, 5% glycerol) at 37 °C. pAgo (500 nM) was preloaded with guide RNA (200 nM) prior to addition of target DNA (100 nM). Reactions were terminated by denaturing stop solution, resolved by 19% urea-PAGE, stained with SYBR Gold, and visualized using a Typhoon FLA 9500 scanner (GE Healthcare, Chicago, IL, USA).

### 2.3. Cell Culturing

HEK293T cells and primary fibroblasts from a healthy donor were provided by the Moscow Branch of the Biobank “All-Russian Collection of Biological Samples of Hereditary Diseases” at the Research Center for Medical Genetics, Moscow, Russia. The cells were grown in high-glucose DMEM containing 10% bovine calf serum and 1× supplements, including antibiotic–antimycotic mixture, sodium pyruvate, non-essential amino acids, and GlutaMAX. The antibiotic–antimycotic mixture contained penicillin, streptomycin, and amphotericin B at final concentrations of 100 U/mL, 100 μg/mL, and 0.25 μg/mL, respectively. Cultures were maintained in T75 flasks under standard conditions at 37 °C and 5% CO_2_ in a humidified incubator.

### 2.4. Lentiviral Assembly

Lentiviral particles were generated in HEK293T cells using calcium phosphate transfection, as described previously [20]. Briefly, cells were plated in 6-well plates and transfected at approximately 80% confluency with the lentiviral transfer vector, psPAX2 packaging plasmid (Addgene #12260, Watertown, MA, USA), and pVSV-G envelope plasmid (Addgene #138479, Watertown, MA, USA). For each well, 5 μg of total plasmid DNA was used to form DNA–calcium phosphate precipitates in 2× HEPES-buffered saline, which were then added dropwise to the cells. After 4–6 h, the precipitate-containing medium was removed and cells were returned to standard culture conditions using serum-supplemented medium prepared with FBS inactivated at 56 °C. To stimulate lentiviral particle production, sodium butyrate was introduced 16 h later at 2 mM. Viral supernatants were collected 48 h later, clarified by low-speed centrifugation, filtered through 0.45 μm PVDF filters (Merck Millipore, Darmstadt, Germany), aliquoted, and stored at −80 °C. HEK293T cells were transduced with lentiviral preparations at MOI 5 in the presence of 8 μg/mL polybrene. Following a 6 h incubation period, the viral medium was removed and replaced with fresh growth medium. Functional lentiviral titers were determined 48 h later by flow cytometric quantification of GFP-expressing cells.

### 2.5. Transfection of Guide RNA and Guide DNA

HEK293T cells were seeded onto gelatin-coated 48-well plates at a density of 7 × 10^4^ cells per well 24 h prior to transfection to achieve approximately 50% confluency. Guide RNAs (or DNAs) were delivered using either Lipofectamine 2000 (Thermo Fisher Scientific, Waltham, MA, USA) or a mitochondria-targeting cationic peptide (Pep1; GLS Biochem, Dalian, China).

For Lipofectamine-mediated delivery, 500 ng of guide RNA (or DNA) was diluted in 25 μL Opti-MEM and combined with 1 μL Lipofectamine 2000 diluted in 25 μL Opti-MEM according to the manufacturer’s instructions. Complexes were incubated for 20 min at room temperature and added dropwise to cells in 48-well plates. Cells were incubated for 48 h prior to downstream analysis.

For visualization experiments using Alexa Fluor 488-labeled guide RNAs, human fibroblasts were used instead of HEK293T cells due to their larger size and more extended mitochondrial network, enabling improved assessment of mitochondrial localization. Cells were seeded onto 48-well plates at a density of 1 × 10^4^ cells per well 24 h prior to transfection to achieve approximately 50% confluency. For peptide-mediated delivery, guide RNAs were diluted in 25 μL Opti-MEM (Mix A) and added to Pep1 (3.2 μg per well) diluted in 25 μL Opti-MEM (Mix B), followed by incubation for 20 min at room temperature to allow for electrostatic complex formation. The resulting RNA–peptide complexes were then added to cells, and live-cell fluorescence imaging was performed at 24 and 48 h post-transfection. Complexes were assembled at an N/P ratio of 2, calculated based on the number of positively charged residues in the peptide (MSLTSSSSSVRVEWIAAVTIAAGTAAIGYLAYK-(RH)_9_-NH_2_) and the phosphate groups of the guide RNA, as described previously [25].

### 2.6. Cytotoxicity Test

Cytotoxicity was evaluated using a resazurin-based viability assay (Abisense, Moscow, Russia) 48 h after lentiviral transduction. Cells were then dissociated, counted, and re-seeded into 96-well plates at 50% confluency equal density per well. After allowing cells to attach, resazurin 1X working solution was added to each well according to the manufacturer’s protocol (incubation at 37 °C in 5% CO_2_ for 4 h). Fluorescence was measured on a multimode plate reader (CLARIOstar, BMG LABTECH, Ortenberg, Germany). Background fluorescence (only medium with resazurin) was subtracted. Cell viability was expressed as fluorescence normalized to the matched control condition (set to 100%).

### 2.7. mtDNA Copy Number Quantification

Total genomic DNA was isolated from HEK293T using the ExtractDNA Blood & Cells Kit (Evrogen, Moscow, Russia) according to the manufacturer’s protocol. mtDNA copy number was determined by quantitative PCR with primer pairs specific for the mitochondrial D-loop or *MT-ND1* loci, while the nuclear *B2M* gene served as an internal normalization control. Amplification reactions were carried out using SYBR Green I master mix (Evrogen, Moscow, Russia) on a QuantStudio 5 Real-Time PCR system (Applied Biosystems, Waltham, MA, USA). Relative mtDNA copy number was calculated using the 2^−ΔΔCt^ method after normalization to *B2M* and control samples as described previously [20]. Each condition included three biological replicates with technical triplicates. Primer sequences are listed in Supplementary Table S1.

### 2.8. Immunocytochemical Assay

Forty-eight hours after lentiviral transduction, fibroblasts were seeded onto 8-well chamber slides pre-coated with gelatin at a density 3.0 × 10^5^ cells per well 48 h after lentiviral transduction. Mitochondrial staining and immunofluorescence analysis were performed as described previously [16,20]. Briefly, cells were incubated with 100 nM MitoTracker CMXROS (Thermo Fisher Scientific, Waltham, MA, USA) for 30 min at 37 °C, washed with PBS, and fixed with 4% paraformaldehyde for 15 min at room temperature, followed by permeabilization in 0.5% Triton X-100. After blocking with 4% bovine serum albumin (BSA), FLAG-tagged proteins were detected using mouse anti-FLAG antibody (ANTI-FLAG M2, Sigma-Aldrich, St. Louis, MO, USA; 1:500) and Alexa Fluor 488–conjugated goat anti-mouse secondary antibody (Thermo Fisher Scientific; 1:400). Samples were mounted in antifade medium and imaged using an Agilent BioTek Lionheart FX fluorescence microscope (Santa Clara, CA, USA).

Images were processed in ImageJ (v1.54p). Co-localization was evaluated by extracting fluorescence intensity profiles along linear regions of interest (ROIs) to assess spatial overlap between mitochondrial and FLAG signals.

### 2.9. Flow Cytometry

Forty-eight hours after transduction, cells were harvested, resuspended in calcium- and magnesium-free PBS, and analyzed by flow cytometry to determine transduction efficiency and estimate functional viral titers. GFP fluorescence was measured using a CytoFLEX S flow cytometer (Beckman Coulter, Brea, CA, USA). GFP-positive populations were defined relative to non-transduced control cells (10,000 events were recorded per sample). Data were analyzed using CytExpert software (v2.4).

### 2.10. Statistical Analysis

Data are presented as mean ± SD from three independent biological replicates, each measured in technical triplicates. Technical replicates were averaged prior to statistical analysis. Statistical significance was assessed using two-tailed Student’s *t*-tests implemented in Python (v3.10.9). Significance levels are indicated as ns (*p* > 0.05), * *p* ≤ 0.05, and ** *p* < 0.01; *** *p* < 0.001.

## 3. Results

### 3.1. Import of pAgos into Human Mitochondria

**Figure 1:**
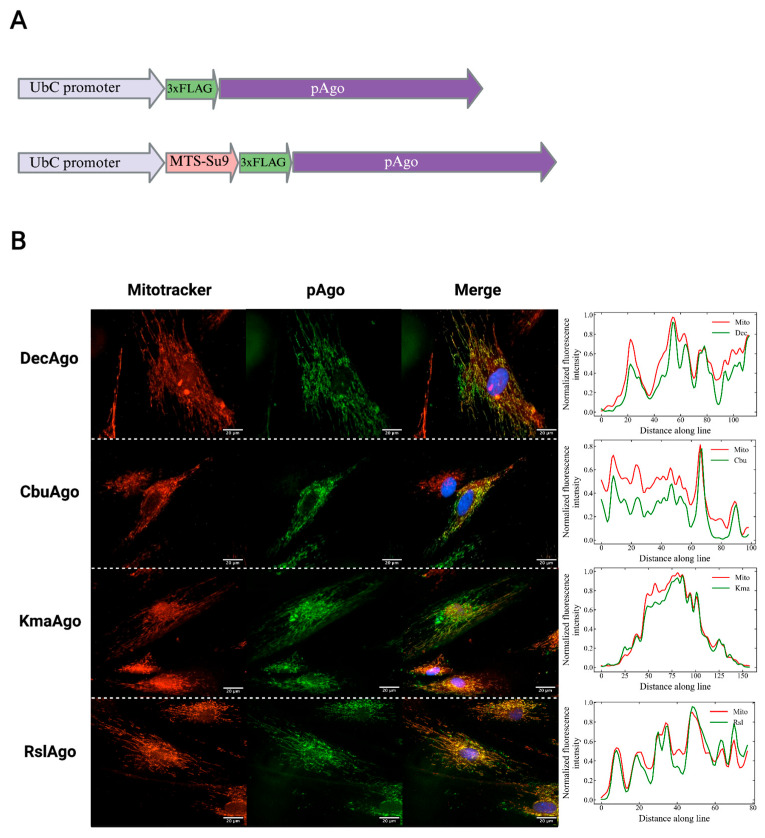
Immunofluorescence analysis of Su9-mediated mitochondrial import of pAgos in human fibroblasts. (**A**)—Schematic representation of lentiviral expression constructs used in this study. pAgos were expressed from the UbC promoter either without a targeting sequence or fused at the N-terminus to the MTS from ATP synthase subunit 9 (MTS-Su9), followed by a 3×FLAG tag for detection. (**B**)—Mitochondrial localization of Su9-3×FLAG-tagged pAgos in human fibroblasts. Representative fluorescence images showing co-localization of pAgos (green) with mitochondria stained with MitoTracker (red) for DecAgo, CbuAgo, KmaAgo, and RslAgo. Nuclei are shown in blue (DAPI). Merged images indicate efficient mitochondrial targeting. Right panels show line-scan intensity profiles demonstrating overlap between pAgo and mitochondrial signals. Quantitative co-localization analysis yielded Pearson’s correlation coefficients ≥ 0.95 for all tested pAgos.

For mitochondrial targeting of pAgo proteins, the Su9 mitochondrial targeting sequence from *Neurospora crassa* was selected as previously described in our earlier study [16,20]. Lentiviral expression constructs were generated in which pAgos were expressed from the UbC promoter either without or with an N-terminal MTS, followed by a 3×FLAG tag (Figure 1A). In the absence of an MTS, pAgos displayed diffuse localization across the cytoplasm and nucleus (Supplementary Figure S1). In contrast, expression of MTS-tagged pAgos in human fibroblasts resulted in clear mitochondrial localization, indicating that mitochondrial import requires an N-terminal targeting signal (Figure 1B). A comparative in silico analysis of alternative MTSs supporting this choice is provided in Supplementary Tables S3–S6.

### 3.2. Evaluation of Guide-Dependent Nuclease Activity of pAgos In Vitro

**Figure 2:**
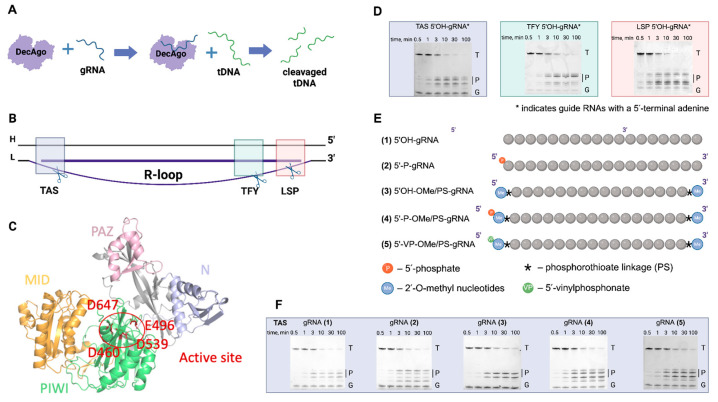
In vitro characterization of DecAgo catalytic activity with the mitochondrial D-loop substrate. (**A**)—Schematic representation of the in vitro cleavage assay. DecAgo was pre-incubated with guide RNA (gRNA) and subsequently combined with a synthetic DNA substrate (target DNA, tDNA), resulting in guide-dependent cleavage of the target DNA. (**B**)—Schematic of the mitochondrial D-loop region indicating target sites used for guide design. Guide RNAs were designed against three regions (TAS, TFY, and LSP). (**C**)—Structural model of DecAgo generated using AlphaFold3, showing canonical Argonaute domains (N, PAZ, MID, and PIWI). Conserved catalytic residues within the PIWI domain are highlighted (red circle). (**D**)—Analysis of DecAgo-mediated cleavage of the D-loop substrate. Representative gel electrophoresis images showing cleavage at TAS, TFY, and LSP sites using different guide RNAs. Progressive depletion of the full-length substrate (T) and accumulation of cleavage products (P) indicate efficient and time-dependent nuclease activity. (**E**)—Schematic representation of guide RNA variants used in the study. Guides include unmodified RNA, 5′-phosphorylated RNA, and chemically modified variants containing 2′-O-methyl nucleotides and terminal phosphorothioate linkages. (**F**)—Cleavage of a DNA target using modified guide RNAs. Positions of the full-length substrate (T) and cleavage products (P) are indicated.

Prior to in vivo experiments, we evaluated the catalytic activity of pAgos in vitro using synthetic guide RNAs or DNAs and a chemically synthesized DNA substrate corresponding to the mitochondrial D-loop region (Figure 2A). The guides were designed against three regions of the D-loop: Termination-Associated Sequence (TAS), Transcription Factor Y-binding site (TFY), and Light Strand Promoter (LSP). For each region, multiple guides complementary to each strand were tested (shown in Figure 2B for the L-strand and Supplementary Figure S2 for the H-strand; see Supplementary Table S2 for all guide sequences).

First, we tested the activity of DecAgo in reactions with various 5′-phosphorylated guide RNAs and target DNA (Figure 2C). Gel electrophoresis analysis revealed robust and time-dependent cleavage of target DNA by DecAgo across all tested regions, as indicated by progressive depletion of the full-length substrate (T) and accumulation of cleavage products (P) (Figure 2D). Cleavage was observed for all three D-loop regions (TAS, TFY, LSP), indicating that DecAgo efficiently processes various target sites within this regulatory locus.

To evaluate the impact of guide chemistry on activity, a series of guide RNAs (H-strand TAS guides) with increasing levels of chemical modification was tested (Figure 2E). An unmodified guide RNA with 5′-OH end served as a control, while subsequent variants incorporated a 5′ phosphate (or 5′-vinylphosphonate) and combinations of stabilizing modifications, including 2′-O-methyl nucleotides and terminal phosphorothioate linkages (Figure 2E, Supplementary Table S2). DecAgo retained cleavage activity across all tested guide variants, but the positions of cleavage varied depending on the structure of the guide 5′ end, in particular, the presence of a 5′-phosphate group (Figure 2F).

**Figure 3:**
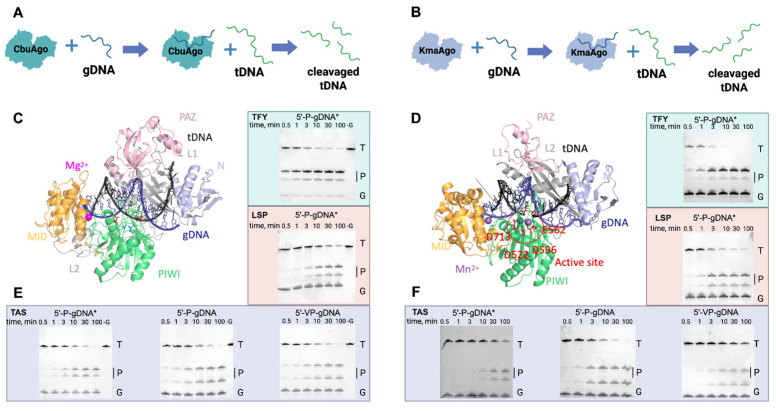
In vitro characterization of CbuAgo and KmaAgo catalytic activity on the mitochondrial D-loop substrate. (**A**,**B**)—Schematic representation of in vitro cleavage assays for CbuAgo (**A**) and KmaAgo (**D**), illustrating guide DNA (gDNA)-dependent targeting and cleavage of double-stranded DNA substrates. (**C**,**D**)—Structural models of CbuAgo (**C**) and KmaAgo (**D**) highlighting the canonical Argonaute domain organization (N, PAZ, MID, and PIWI) and conserved catalytic residues within the PIWI domain. The CbuAgo model is based on the crystal structure described previously (6QZK; [26]). The KmaAgo model is based on the experimentally determined structure (8XK3; [13]). (**E**,**F**)—Analysis of CbuAgo (**E**)- and KmaAgo (**F**)-mediated cleavage of the R-loop substrate. Representative gel electrophoresis images showing cleavage at TAS, TFY, and LSP sites using three unmodified distinct 5′-phosphorylated guide DNAs (5′-P-gDNAs*) with 5′-terminal nucleotide adenine, designed to target the corresponding loci. In addition, two guide DNAs starting with thymine were used, carrying either a 5′-phosphate or a 5′-vinylphosphonate modification (5′-P-gDNA and 5′-VP-gDNA).

We further evaluated the in vitro activity of CbuAgo and KmaAgo, which utilize DNA guides (gDNAs) and cleave DNA targets (Figure 3A–D). In these assays, pAgo proteins were pre-incubated with mtDNA-specific guide DNAs and subsequently combined with synthetic DNA corresponding to the mitochondrial D-loop region. Both proteins efficiently cleaved the target DNA strand with TAS, TFY and LSP guide DNAs (5′-P-gDNA*; Figure 3E,F).

To assess the effect of guide chemistry on cleavage activity, two additional guide variants (H-strand TAS guides) with another sequence, containing a 5′-thymine instead of adenine present in all other guides (5′-P-gDNA) and a chemically modified guide DNA bearing a 5′-vinylphosphonate (5′-VP-gDNA) were used. Gel electrophoresis analysis demonstrated that both CbuAgo and KmaAgo exhibited guide-dependent cleavage activity across all three tested regions (TAS, TFY, and LSP), as evidenced by progressive reduction in the full-length substrate and accumulation of cleavage products (Figure 3E,F).

In contrast to DecAgo, CbuAgo and KmAgo, which cleave DNA targets, RslAgo is a DNA-guided RNA nuclease. Its in vitro activity has been previously analyzed in detail in a previous study and therefore was not re-evaluated here [16].

### 3.3. Effect of pAgos on mtDNA Copy Number in HEK293T Cells

**Figure 4:**
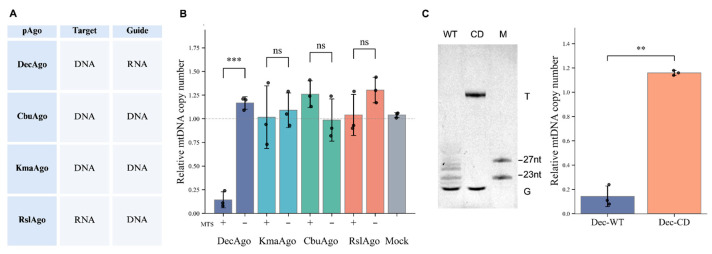
Analysis of the mtDNA copy number following expression of pAgos in HEK293T cells. (**A**)—Summary of target and guide specificities of the pAgos used in this study. (**B**)—Quantification of relative mtDNA copy number in HEK293T cells expressing the indicated pAgos with corresponding guides (RNA or DNA) with or without mitochondrial targeting sequence (MTS). A significant reduction in mtDNA copy number is observed only for MTS-targeted DecAgo, whereas other pAgos do not produce measurable effects. A mock control containing only the 5′OH-gRNA* targeting the TAS region of the mitochondrial heavy strand (H-strand) did not result in detectable mtDNA depletion. Data are represented as mean ± SD (*n* = 3 technical replicates from 3 biological replicates). Statistical significance was assessed using an unpaired two-tailed Student’s *t*-test. The dashed line indicates the reference baseline of 1, corresponding to the average mtDNA copy number in untreated HEK293T cells (HEK293T-UT), used for relative quantification. (**C**)—In vitro cleavage assay comparing wild-type DecAgo (WT) and catalytically inactive mutant (DecAgo-CD). Quantification of relative mtDNA copy number confirms that only catalytically active DecAgo induces mtDNA reduction. Data are represented as mean ± SD (*n* = 3 technical replicates from 3 biological replicates). Statistical significance was assessed using an unpaired two-tailed Student’s *t*-test: ns, not significant (*p* > 0.05); ** *p* < 0.01; *** *p* < 0.001.

To assess the in vivo activity of pAgo proteins, HEK293T cells were transduced with lentiviral constructs encoding each of pAgos. While DecAgo, CbuAgo and KmaAgo all target DNA substrates, RslAgo was included to test whether targeting RNA substrates, such as R-loops, could also affect mtDNA maintenance. DNA or RNA guides corresponding to mtDNA were co-transfected according to the pAgo used (Figure 4A). For DecAgo, a guide RNA targeting the TAS region of the mitochondrial heavy strand (H-strand) and bearing a 5′-terminal adenine (5′OH-gRNA*) was used. For CbuAgo and KmaAgo, a guide DNA targeting the TAS region complementary to the H-strand (5′-P-gDNA) was used for mtDNA copy number analysis. For RslAgo, in vivo experiments were performed using a guide DNA targeting the TAS region of the light strand (L-strand) (5′-P-gDNA*). Based on the hypothesis that the single-stranded region of the mitochondrial D- or R-loop represents a vulnerable substrate for pAgo-mediated cleavage, mtDNA copy number was quantified by quantitative real-time PCR relative to untreated control cells (UT) as a functional readout of mitochondrial genome stability (Figure 4B).

A pronounced reduction in mtDNA levels (~5-fold) was observed only in cells expressing DecAgo fused to the MTS-Su9 sequence, whereas no significant changes were detected for KmaAgo, CbuAgo, or RslAgo, under any tested conditions, including in the absence of exogenous guides (Supplementary Figure S3). Notably, the effect of DecAgo was independent of exogenously supplied guide RNAs, as comparable mtDNA depletion was observed in both the presence and absence of guides (Supplementary Figure S3). These results indicate that DecAgo remains active in mitochondria under cellular conditions, whereas the other tested pAgos do not measurably affect mtDNA maintenance despite efficient mitochondrial localization. This difference may reflect their reliance on DNA guides rather than RNA guides (see Discussion).

To determine whether the observed effect of DecAgo depends on its catalytic activity, we generated its PIWI-domain mutant carrying D460A and D539A substitutions (DecAgo-CD, for catalytically dead). In contrast to wild-type DecAgo, the DecAgo-CD mutant did not exhibit cleavage activity in vitro and likewise did not result in any detectable reduction in mtDNA copy number in cells (Figure 4C). These results indicate that mtDNA depletion requires PIWI-mediated catalytic activity and support the hypothesis that endogenous mitochondrial nucleic acids may serve as functional guides in this context.

### 3.4. Peptide-Mediated Import of Fluorescent Guide RNAs into Mitochondria

Experiments described above demonstrated that DecAgo could efficiently decrease the mtDNA copy number but that this effect was independent of exogenous guide RNAs. To improve mitochondrial delivery of exogenous guide RNAs, we adapted a peptide-based strategy inspired by a previously described mitochondria-targeting carrier derived from mitoNEET, a small outer mitochondrial membrane protein involved in cellular redox regulation [25]. Based on this design, we used a chimeric peptide comprising the N-terminal targeting sequence of mitoNEET fused to a C-terminal cationic (RH)9 segment (mitoNEET-(RH)9; see Section 2.5 for sequence details). The N-terminal region contains a membrane-targeting sequence that is expected to promote mitochondrial localization, whereas the arginine/histidine-rich segment provides a high positive charge density, enabling electrostatic interaction with the negatively charged phosphate backbone of the guide RNA and formation of peptide–RNA complexes. Peptide–RNA complexes were assembled with Alexa Fluor 488-labeled guide RNA (Supplementary Table S2) at an N/P ratio of 2, which was empirically determined in our experiments to provide optimal complex formation and delivery efficiency.

**Figure 5:**
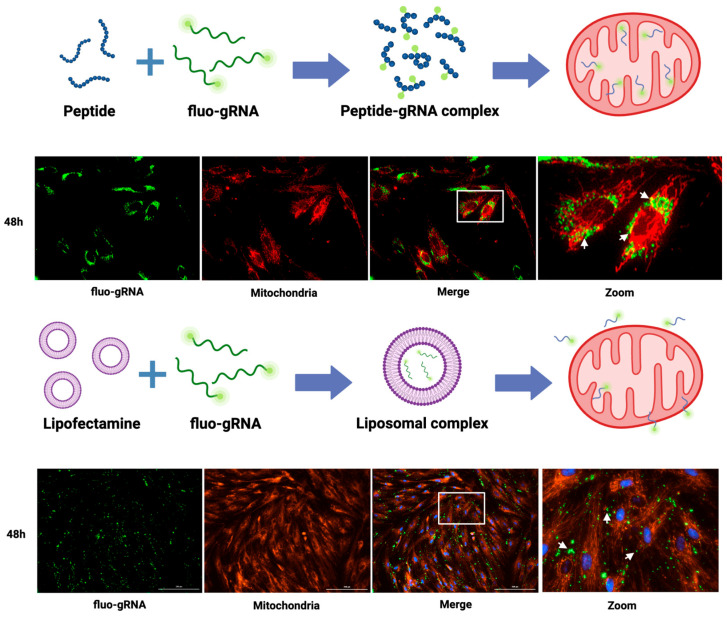
Live-cell imaging of peptide-mediated mitochondrial delivery (**top**) and Lipofectamine-mediated transfection (**bottom**) of fluorescent guide RNAs at 48 h into human fibroblasts. The white rectangles indicate the regions enlarged in the corresponding Zoom panels. White arrows mark representative sites of mitochondrial localization of fluorescent guide RNAs.

Live-cell fluorescence imaging using widefield microscopy revealed improved co-localization of peptide-delivered fluorescent guide RNAs with mitochondria compared to Lipofectamine-mediated transfection, indicating enhanced mitochondrial targeting efficiency (Figure 5). However, delivery of guide RNAs using this approach did not result in detectable changes in mtDNA copy number with DecAgo under our experimental conditions (Supplementary Figure S4). Given that DecAgo-dependent mtDNA depletion was observed even in the absence of exogenous guides, this result is consistent with the possibility that endogenous mitochondrial nucleic acids may dominate guide loading in cells and thereby mask the contribution of externally supplied guide RNAs.

## 4. Discussion

In this study, we showed that pAgo proteins of various classes can be efficiently targeted to human mitochondria using the Su9 mitochondrial targeting sequence. All tested enzymes were successfully imported and did not exhibit detectable cytotoxicity 48 h after transduction (Supplementary Figure S5). However, only DecAgo exhibited detectable functional activity in cells, resulting in an approximately fivefold reduction in mtDNA copy number. Our results indicate that efficient mitochondrial import of pAgos alone is not sufficient to induce mtDNA depletion in cells, and suggest that the major limiting factor is likely the availability and mitochondrial delivery of compatible guides.

Indeed, DecAgo-mediated depletion of mtDNA occurred independently of exogenously supplied guide RNAs. This effect was abolished in a catalytically inactive DecAgo variant carrying mutations in the PIWI domain, indicating that mtDNA reduction is directly linked to its nuclease activity. Together, these findings suggest that DecAgo remains active in mitochondria and may utilize endogenous nucleic acids as guide molecules. This interpretation is consistent with growing evidence that mitochondria contain a diverse population of endogenous RNA species, including transcripts originating from the D-loop region, antisense RNAs and processed noncoding RNAs [11]. Although the functional roles of many of these molecules remain incompletely understood, their presence provides a plausible source of guides that could serve as guide molecules for RNA-guided nucleases. However, the identity and functional properties of these endogenous guides remain unclear and will require further investigation.

In contrast, CbuAgo and KmaAgo did not produce measurable effects on mtDNA copy number in vivo, despite exhibiting robust cleavage activity in vitro. A likely explanation lies in their strict dependence on DNA guides. In contrast to RNA import, which has been partially characterized, efficient import of short DNA molecules into mitochondria remains poorly understood, although recent studies suggest that chemically modified DNA molecules may enter mitochondria under specific experimental conditions [27]. Most reported approaches rely on engineered delivery systems, such as mitochondria-targeted nanoparticles or protein-based carriers, rather than endogenous transport mechanisms, underscoring a major limitation for DNA-guided genome editing strategies in mitochondria [26]. This reflects the well-known difficulty of nucleic acid delivery into mitochondria. Without efficient guide import and the absence of endogenous single-stranded DNA oligonucleotides, DNA-guided Argonautes are unlikely to be active in mitochondria.

RslAgo represents a distinct case, as it preferentially targets RNA substrates. In this study, we attempted to exploit this property by designing DNA guides against the mitochondrial R-loop region. However, this approach did not result in detectable changes in mtDNA copy number. At present, it is not possible to determine the exact reason for this lack of effect. One possibility is that the required DNA guides were not efficiently delivered into mitochondria (see above). Alternatively, RslAgo may not efficiently act on R-loop structures under these conditions. Since RslAgo was successfully localized to mitochondria, the lack of a detectable effect on mtDNA copy number is unlikely to be caused by protein delivery. Finally, we note that our analysis focused on mtDNA copy number as a readout and did not assess mitochondrial transcript levels. It is therefore possible that RslAgo activity, if present in eukaryotic cells, may affect RNA species rather than DNA maintenance. Exploring such transcript-level effects falls beyond the scope of the current study but represents an important direction for future work.

To improve mitochondrial delivery of guide molecules, we tested a peptide-based strategy for the transport of fluorescently labeled guide RNAs. This approach resulted in enhanced co-localization of guides with mitochondria compared to Lipofectamine-mediated transfection, indicating improved targeting at the cellular level. We next assessed the functional impact of guide delivery by measuring mtDNA copy number following transfection of both RNA and DNA guides for the corresponding pAgos. In the case of DecAgo, mtDNA copy number was reduced to a similar extent regardless of whether guides were delivered by Lipofectamine or by peptide or not supplied at all. This suggests that the contribution of externally delivered guides is limited under these conditions, potentially due to non-specific activity or preferential use of endogenous nucleic acids.

Potential off-target activity remains an important consideration for programmable nuclease systems, particularly in light of recent studies highlighting safety challenges associated with CRISPR-based genome editing technologies, including unintended cleavage and large-scale genomic alterations [28]. Unlike CRISPR-Cas nucleases, however, the pAgos investigated in this study introduce single-stranded cleavage events in DNA/RNA substrates under physiological conditions rather than double-stranded DNA breaks, potentially limiting activity toward fully double-stranded genomic regions. Consistent with this idea, in our previous work with mitochondria-targeted AmAgo, guides directed against double-stranded mtDNA regions did not induce detectable mtDNA depletion [20]. Nevertheless, comprehensive characterization of potential off-target effects will be essential for future development of pAgo-based mitochondrial editing systems.

Taken together, our results identify RNA-guided DecAgo as an active nuclease in human mitochondria and suggest that endogenous mitochondrial nucleic acids may support its activity in the absence of externally delivered guides. Previously, we have shown that another RNA-guided pAgo, AmAgo, is also capable of reducing mtDNA copy number, indicating that RNA-guide-dependent pAgos may be more suited for mtDNA engineering [20]. At the same time, they highlight that guide delivery remains a key limitation for extending this approach to other Argonautes. Future work should focus on improving the mitochondrial delivery of short DNA guides, as well as increasing the specificity of DecAgo and AmAgo toward externally supplied RNA guides, for example through optimized guide design or delivery as pre-assembled ribonucleoprotein complexes (RNP). Defining the identity of endogenous guide molecules and understanding their contribution to Argonaute activity will also be essential for the development of programmable mitochondrial genome editing strategies.

## Data Availability

The original contributions presented in this study are included in the article/Supplementary Materials. Further inquiries can be directed to the corresponding authors.

## Supplementary Materials

The following supporting information can be downloaded at: https://www.mdpi.com/article/10.3390/cells15121129/s1, Table S1: Primer sequences; Table S2: Guide RNA/DNA and DNA matrix sequences used for in vitro assay; Figure S1: Immunofluorescence analysis of DecAgo without an MTS; Tables S3–S6: Physicochemical properties of mitochondrial targeting sequences (MTS) fused to pAgos; Figure S2: In vitro cleavage of the mtDNA D-loop TAS region (H-strand) by pAgos using guide RNAs differing in 5′-end chemistry; Figure S3: Relative mtDNA copy number was measured in HEK293T cells expressing pAgos in the presence or absence of exogenous guide RNAs/DNAs; Figure S4: Relative mtDNA copy number was measured in HEK293T cells expressing DecAgo-WT following delivery of guide RNA either using Lipofectamine 2000 (DecAgo–Pep) or in combination with peptide-mediated delivery (DecAgo + Pep); Figure S5: Cell viability was evaluated using a resazurin-based assay and is presented as fluorescence normalized to untreated control cells (UT, 100%).
