# Supplementary material for: Activity of DNA- and RNA-Guided Prokaryotic Argonautes in Human Mitochondria
Source: Cells. 2026 Jun 22;15(12):1129. doi: 10.3390/cells15121129 (PMC13297701; doi:10.3390/cells15121129)
Supplement: Supplementary file 1 [file cells-15-01129-s001.zip › cells-4328148-supplementary.pdf]

Supplementary data

Table S1. Primer sequences. List of oligonucleotide primers used for qPCR-based mtDNA quantification (e.g., B2M, D-loop, ND1) and for molecular cloning of constructs (e.g., Dec/Cbu/Kma/Rsl agos, Su9, FLAG). Sequences are provided in 5′ to 3′ orientation.

Primer	Sequence
B2M_F	TGCTGTCTCCATGTTTGATGTATCT
B2M_R	TCTCTGCTCCCCACCTCTAAGT
D-loop_F	CCTAACACCAGCCTAACCAGATTTC
D-loop_R	AGATTAGTAGTATGGGAGTGGGAGG
ND1_F	CCCTAAAACCCGCCACATCT
ND1_R	GAGCGATGGTGAGAGCTAAGGT
GAG_F	TCTCGACGCAGGACTCG
GAG_R	TACTGACGCTCTCGCACC
DecClon_F	AATTCTCGAGATGTTGATCTCCAAACCAATACCGTG
DecClon_R	AATTGCTAGCTTAACCTTCTCCGCGTGTGTTGG
CbuClon_F	TTAAGGATCCATGAATAATCTTACTTTCGAAGCGTTCG
CbuClon_R	TTAAGCTAFCTCAGAGGAAAAAAAGTCTGTTATCTACG
KmaClon_F	ATTCTCGAGATGGAGGCGTACATAACCGAGATG
KmaClon_R	ATTGCTAGCTCAAACAAACGGAAGATTGTTTGGTTACC
RslClon_F	AATCTCGAGATGACCCGCTACGAAACTAACATC
RslClon_R	AATTGCTAGCTCAAAGCATTTCATCCTTAAGGATTTCTG C
Su9_KmaRslCbu_clon_R	AATGCTAGCATCTCGAGCTTGTCATCGTCATCCTTGTAA TC
UbC_F	GCTGAAGCTCCGGTTTTGAACT
WPRE_R	CATAGCGTAAAAGGAGCAACA
Su9_pUltra_clon_F	ACCGGTGCCACCATGGCCTCCACTCGTGTCCT
Su9_pUltra_clon_R	AATGCTAGCATCTCGAGCTTGTCATCGTCATCCTTGTAA TC

FLAG_pUltra_clon_F	AATTACCGGTGCCACCATGGACTACAAAGACCATGAC GGTGAT

Table S2. Guide RNA/DNA and DNA matrix sequences used for in vitro assay.

**Name**	**mtDNA site**	**Sequence**	**Comments**
**guide RNA/DNA**			
5′OH-gRNA*	H-strand TAS guide RNA	AGUACAUAAAAACCCAAU	ssRNA guide for Dec
5′OH-gRNA	H-strand TAS guide 2 RNA	UGUAGAGUACAUAAAAA C	ssRNA guide for Dec
5′-P-gRNA	H-strand TAS guide 2m RNA	p- UGUAGAGUACAUAAAAA C	ssRNA guide for Dec
5′OH-OMe/PS-gRNA	H-strand TAS guide 2m1 RNA	mU*GUAGAGUACAUAAAA A*mC	ssRNA guide for Dec
5′-P-OMe/PS-gRNA	H-strand TAS guide 2m2 RNA	p- mU*GUAGAGUACAUAAAA A*mC	ssRNA guide for Dec
5′-VP-OMe/PS-gRNA	H-strand TAS guide 2m3 RNA	pv- mU*GUAGAGUACAUAAAA A*mC	ssRNA guide for Dec
5′OH-gRNA*	L-strand TAS guide RNA	AUUGGGUUUUUAUGUAC U	ssRNA guide for Dec
5′OH-gRNA*	H-strand TFY guide RNA	ACAUCAUAACAAAAAAU U	ssRNA guide for Dec
5′OH-gRNA*	L-strand TFY guide RNA	AAUUUUUUGUUAUGAUG U	ssRNA guide for Dec
5′OH-gRNA*	H-strand LSP guide RNA	AUUUUAUCUUUUGGCGG U	ssRNA guide for Dec
5′OH-gRNA*	L-strand LSP guide RNA	ACCGCCAAAAGAUAAAAU	ssRNA guide for Dec

5′-P-gDNA*	H-strand TAS guide DNA	pAGTACATAAAAACCCAAT	ssDNA guide for Cbu/Kma/Rsl
5′-P-gDNA	H-strand TAS guide 2 DNA	pTGTAGAGTACATAAAAAC	ssDNA guide for Cbu/Kma/Rsl
5′-VP-gDNA	H-strand TAS guide 2m DNA	pv-UGTAGAGTACATAAAAAC	ssDNA guide for Cbu/Kma/Rsl
5′-P-gDNA*	L-strand TAS guide DNA	pATTGGGTTTTTATGTACT	ssDNA guide for Cbu/Kma/Rsl
5′-P-gDNA*	H-strand TFY guide DNA	pACATCATAACAAAAAATT	ssDNA guide for Cbu/Kma/Rsl
5′-P-gDNA*	L-strand TFY guide DNA	pAATTTTTTGTTATGATGT	ssDNA guide for Cbu/Kma/Rsl
5′-P-gDNA*	H-strand LSP guide DNA	pATTTTATCTTTTGGCGGT	ssDNA guide for Cbu/Kma/Rsl
5′-P-gDNA*	L-strand LSP guide DNA	pACCGCCAAAAGATAAAAT	ssDNA guide for Cbu/Kma/Rsl
fluo-guide RNA	L-strand TAS guide RNA	AUUGGGUUUUUAUGUACU[Alexa488]	fluo-labeled ssRNA
**matrix for in vitro testing**			
tDNA1	H-strand TAS matrix DNA	CTTGACCACCTGTAGAGTACATAAAAACCCAATCCACATCAAAACCCCCT	target DNA for Dec/Cbu/Kma
tDNA2	L-strand TAS matrix DNA	GGGGTTTTGATGTGGATTGGGTTTTTATGTACTCTACAGGTGGTCAAGTA	target DNA for Dec/Cbu/Kma
tDNA3	H-strand LSP matrix DNA	CTAACCAGATTTCAAATTTTATCTTTTGGCGGTATGCACTTTTAACAGTC	target DNA for Dec/Cbu/Kma
tDNA4	L-strand LSP matrix DNA	CTGTTAAAAGTGCATACCGCCAAAAGATAAAATTTGAAATCTGGTTAGGC	target DNA for Dec/Cbu/Kma

Figure S1. Immunofluorescence analysis of DecAgo without an MTS reveals diffuse cytoplasmic and nuclear distribution with no detectable mitochondrial localization.

DecAgo was visualized using anti-3×FLAG antibodies (green), mitochondria were labeled with MitoTracker (red), and nuclei were stained with DAPI (blue); merged images are shown.

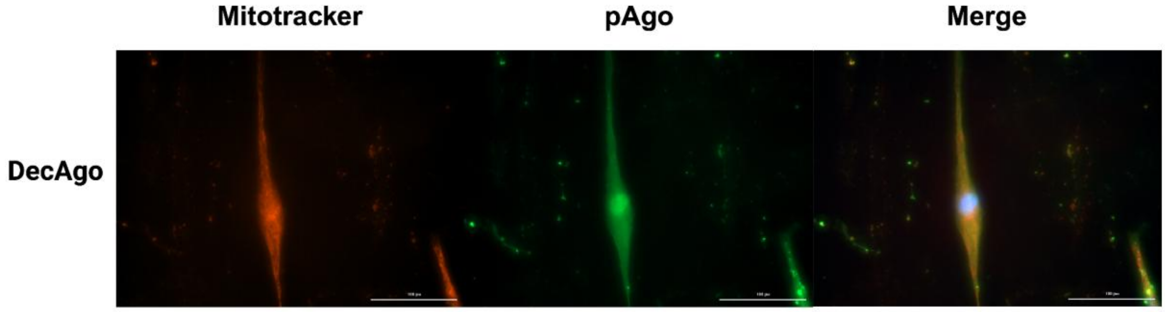

Tables S3-S6. Physicochemical properties of mitochondrial targeting sequences (MTS) fused to pAgos. Summary of calculated parameters for each MTS-pAgo construct, including net charge, isoelectric point (pI), and hydropathy index (GRAVY).

**DecAgo-construct**	**MTS charge**	**MTS pI**	**MTS GRAVY**	**Full charge**	**Full pI**	**Full GRAVY**
SOD2-3xFLAG	+3	10.76	0.163	+4	8.70	-0.390
COX8A-3xFLAG	+5	12.48	0.362	+6	9.01	-0.378
ATG4D-3xFLAG	+10	11.17	-0.337	+5	9.28	-0.406
Su9-3xFLAG	+13	12.55	-0.272	+7	9.01	-0.466

**CbuAgo-construct**	**MTS charge**	**MTS pI**	**MTS GRAVY**	**Full charge**	**Full pI**	**Full GRAVY**
SOD2-3xFLAG	+3	10.76	0.163	+14	8.85	-0.387
COX8A-3xFLAG	+5	12.48	0.362	+16	8.94	-0.376
ATG4D-3xFLAG	+10	11.17	-0.337	+21	9.06	-0.401

Su9-3xFLAG	+13	12.55	-0.272	+24	9.16	-0.393

KmaAgo-construct	MTS charge	MTS pI	MTS GRAVY	Full charge	Full pI	Full GRAVY
SOD2-3xFLAG	+3	10.76	0.163	+21	9.26	-0.361
COX8A-3xFLAG	+5	12.48	0.362	+23	9.34	-0.350
ATG4D-3xFLAG	+10	11.17	-0.337	+28	9.42	-0.375
Su9-3xFLAG	+13	12.55	-0.272	+31	9.52	-0.369

RslAgo-construct	MTS charge	MTS pI	MTS GRAVY	Full charge	Full pI	Full GRAVY
SOD2-3xFLAG	+3	10.76	0.163	+3	7.97	-0.435
COX8A-3xFLAG	+5	12.48	0.362	+5	8.26	-0.425
ATG4D-3xFLAG	+10	11.17	-0.337	+10	8.62	-0.447
Su9-3xFLAG	+13	12.55	-0.272	+13	8.79	-0.439

Figure S2. In vitro cleavage of the mtDNA D-loop TAS region (H-strand) by pAgos using guide RNAs differing in 5′-end chemistry (5′-P-gRNA*, 5′-P-gRNA, and 5′-VP-gRNA). Schematic representation of the mitochondrial D-loop region indicating target sites within

the termination-associated sequence (TAS), transcription factor Y (TFY), and light-strand promoter (LSP) regions of the H-strand (top). Cleavage assays were performed using RNA-guided (DecAgo)) and DNA-guided (CbuAgo and KmaAgo) Argonautes on synthetic DNA substrates corresponding to the indicated regions. Reactions were analyzed over time (0.5–100 min). T, target substrate; P, cleavage products; G, guide (bottom).

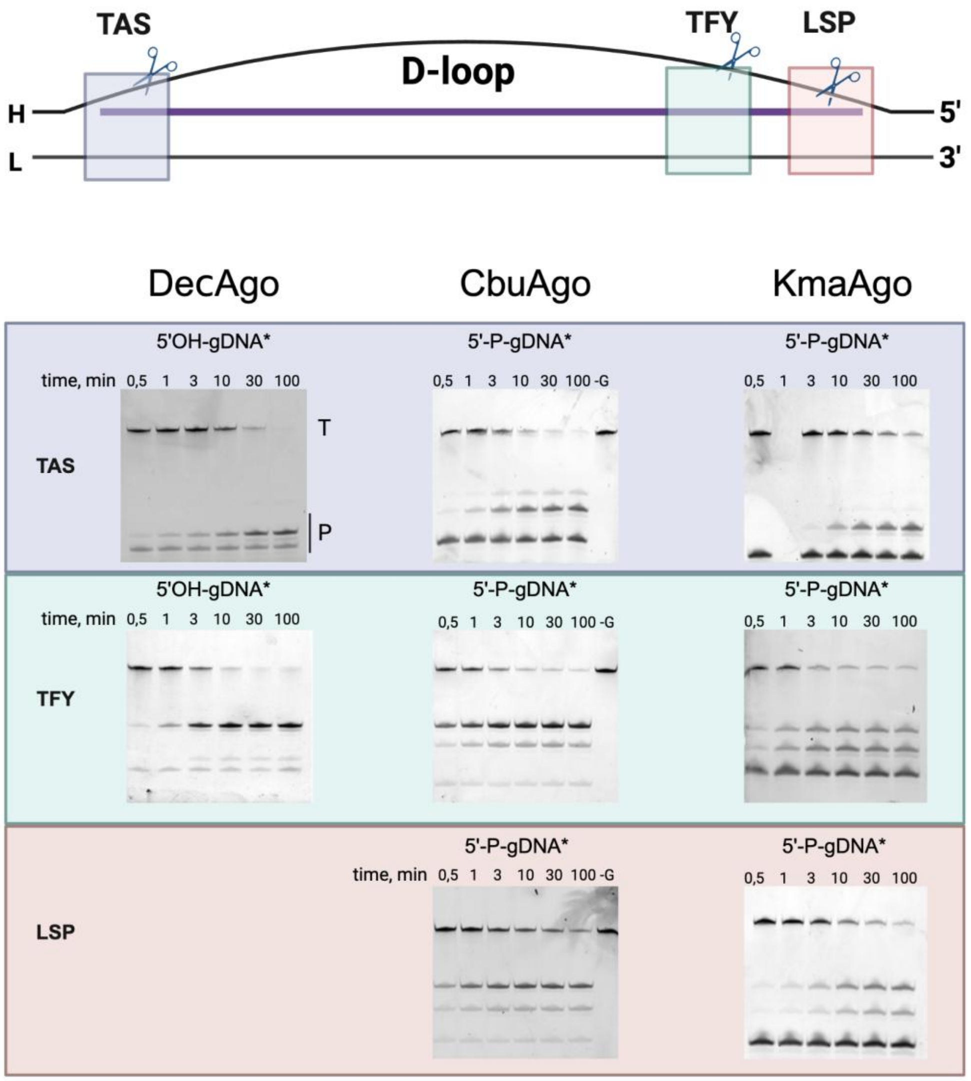

Figure S3. Relative mtDNA copy number was measured in HEK293T cells expressing pAgos in the presence or absence of exogenous guide RNAs/DNAs. Comparable reduction in mtDNA copy number was observed under both conditions, indicating that pAgos activity is independent of externally delivered guides. No statistically significant

differences were detected between the groups ($p > 0.05$, t-test). Data are represented as mean ± SD (n = 3 technical replicates from 3 biological replicates).

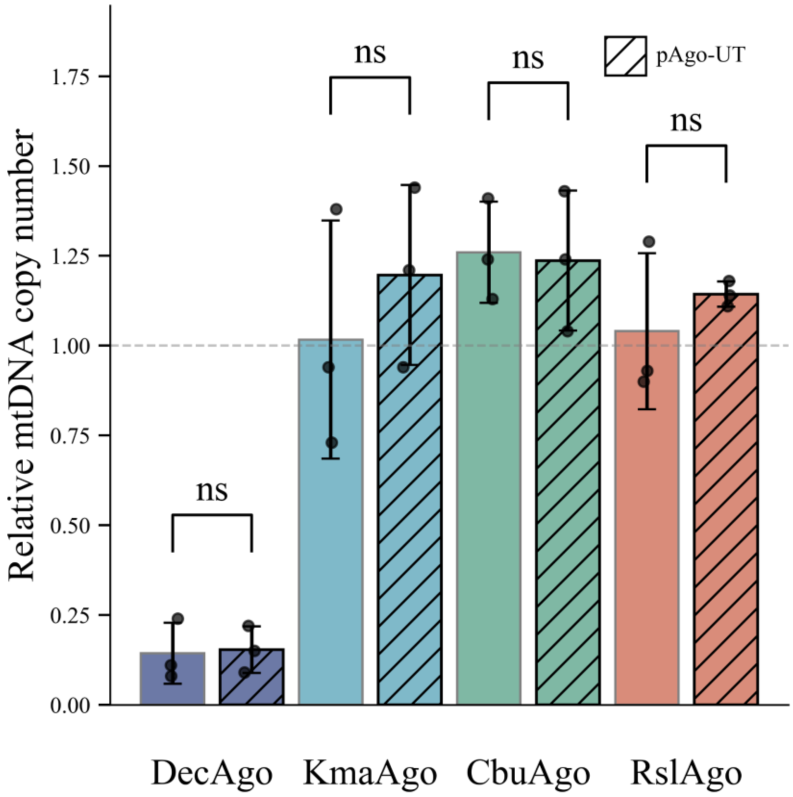

Figure S4. Relative mtDNA copy number was measured in HEK293T cells expressing DecAgo-WT following delivery of guide RNA either using Lipofectamine 2000 (DecAgo−Pep) or in combination with peptide-mediated delivery (DecAgo+Pep). No statistically significant difference between the conditions was observed ($p = 0.237$, t-test). Data are represented as mean ± SD (n = 3 technical replicates from 3 biological replicates).

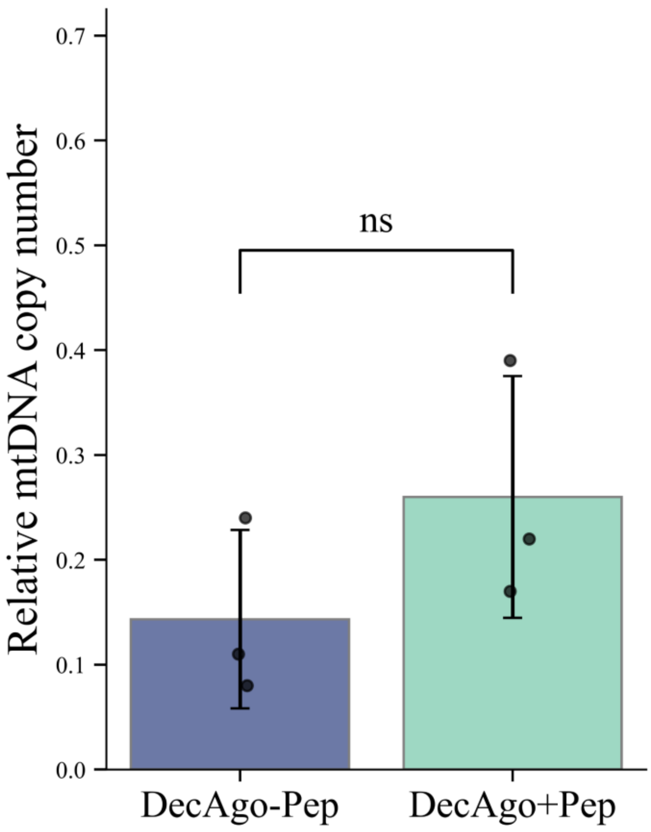

Figure S5. Cell viability was evaluated using a resazurin-based assay and is presented as fluorescence normalized to untreated control cells (UT, 100%). No statistically significant differences in cell viability were observed between pAgo-expressing cells and control ($p >$ 0.05, *t*-test). Data are represented as mean ± SD (n = 3 technical replicates from 1 biological replicate).

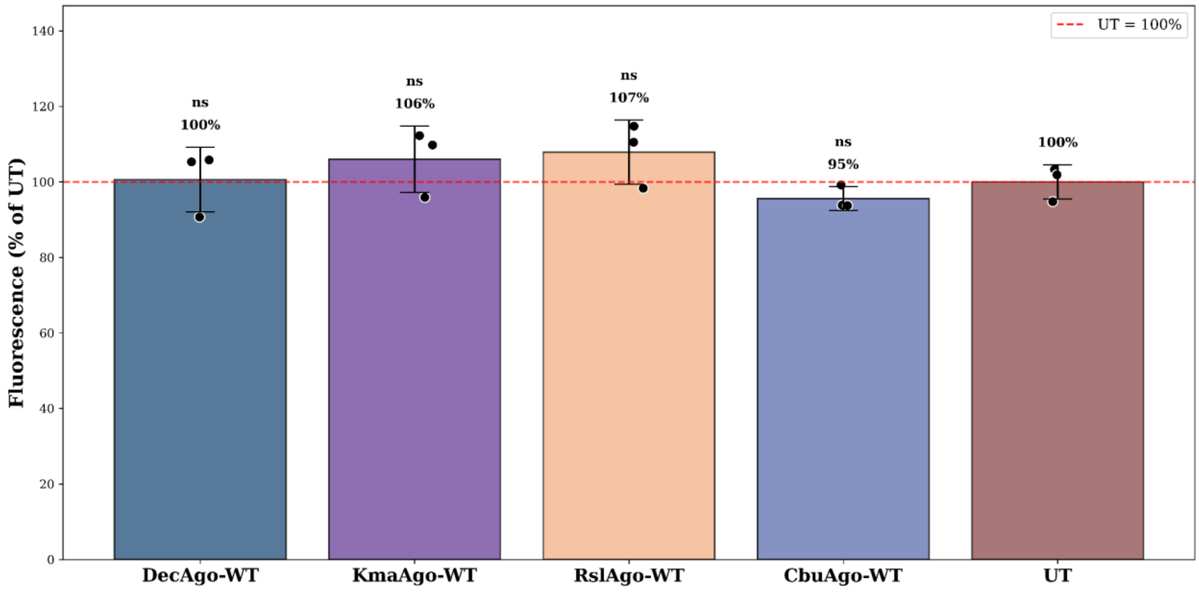
UT = 100%
Fluorescence (% of UT)
140
120
100
80
60
40
20
0
ns
100%
ns
106%
ns
107%
ns
95%
100%
DecAgo-WT
KmaAgo-WT
RslAgo-WT
CbuAgo-WT
UT
